# Recombinant rabies virus particles presenting botulinum neurotoxin antigens elicit a protective humoral response *in vivo*

**DOI:** 10.1038/mtm.2014.46

**Published:** 2014-10-01

**Authors:** Andrew W Hudacek, Fetweh H Al-Saleem, Mallory Willet, Travis Eisemann, Jeffrey A Mattis, Lance L Simpson, Matthias J Schnell

**Affiliations:** 1 Molecular Targeting Technologies, Inc., West Chester, Pennsylvania, USA; 2 Department of Microbiology and Immunology, Sidney Kimmel Medical College, Thomas Jefferson University, Philadelphia, Pennsylvania, USA; 3 Inventox, Inc., Lankenau Institute for Medical Research, Wynnewood, Pennsylvania, USA; 4 Department of Biochemistry, Sidney Kimmel Medical College, Thomas Jefferson University, Philadelphia, Pennsylvania, USA; 5 Jefferson Vaccine Center, Thomas Jefferson University, Philadelphia, Pennsylvania, USA

## Abstract

Botulinum neurotoxins are one of the most potent toxins found in nature, with broad medical applications from cosmetics to the treatment of various neuropathies. Additionally, these toxins are classified as Category A-Tier 1 agents, with human lethal doses calculated at as little as 90 ng depending upon the route of administration. Of the eight distinct botulinum neurotoxin serotypes, the most common causes of human illness are from serotypes /A, /B, and /E. Protection can be achieved by eliciting antibody responses against the receptor-binding domain of the neurotoxin. Our previous research has shown that recombinant rabies virus–based particles can effectively present heterologous antigens. Here, we describe a novel strategy using recombinant rabies virus particles that elicits a durable humoral immune response against the botulinum neurotoxin receptor binding domains from serotypes /A, /B, and /E. Following intramuscular administration of β-propiolactone-inactivated rabies virus particles, mice elicited specific immune responses against the cognate antigen. Administration of a combination of these vectors also demonstrated antibody responses against all three serotypes based on enzyme-linked immunosorbent assay (ELISA) measurements, with minimal decay within the study timeline. Complete protection was achieved against toxin challenge from the serotypes /A and /B and partial protection for /E, indicating that a multivalent approach is feasible.

## Introduction

Botulism is a potentially fatal neuroparalytic illness resulting from intoxication by various species of the Gram-positive spore forming bacterium *Clostridium* genus*. C. botulinum, C. barati, C. butyricum*, and *C. argentiense* can produce eight distinct serotypes of botulinum neurotoxins,^1,2^ A through H, with the genes for BoNT/C and /D encoded by bacteriophage and can be produced as chimeric toxins.^3–6^ These toxins inhibit neuromuscular signaling resulting in neuromuscular weakness and flaccid paralysis,^7^ with death generally resulting from respiratory failure. While the disease can occur naturally, often from contaminated food or wound infections, intoxication could also occur as a result of an act of bioterrorism, with the National Institutes of Health (NIH) classifying *C. botulinum* as a Category A priority pathogen because of the potential for weaponizing BoNT.^5^

The BoNT is a zinc-endopeptidase that is synthesized as an inactive precursor that is processed to form a biologically active disulfide linked di-chain molecule and is secreted in a multiprotein complex ranging between 300 and 900 kDa, depending upon the serotype and *Clostridial* strain.^8^ The disulfide linked di-chain molecule consists of a 50 kDa light chain responsible for endopeptidase activity and a 100 kDa heavy chain (HC) that is involved in receptor binding and translocation of the toxin across the endosomal membrane.^6^ The receptor-binding region (referred to as either heavy chain, receptor binding or H_C_50 (heavy chain, C-terminal 50 kDa fragment)) of the heavy chain binds to synaptogamin I and II on the surface of neurons, and is then endocytosed where the HCT/H_N_50 lectin-like domain mediates translocation across the endosomal membrane and release of the catalytic light chain.^9^ Depending upon the BoNT serotype, the protease targets at least one of three different proteins: VAMP/Synaptobrevin, SNAP-25 or syntaxin.^7^ The most common serotypes responsible for human intoxication are A, B and E,^10^ with serotypes A and E acting upon SNAP-25 and serotype B acting on VAMP/Synaptobrevin.^8^

The current vaccine strategy that is employed by the Department of Defense utilizes formalin-inactivated holotoxin.^11^ However, the supply of this vaccine is limited, expensive to produce and the efficacy is questionable.^5^ Therefore, alternative strategies are being explored that utilize subunits of the toxin, particularly the H_C_50 fragment which are produced in *Escherichia coli*, *Pichia pastoris*, or other systems.^11–17^ The addition of adjuvants when administering recombinant purified H_C_50 proteins has been shown to enhance the immune response,^18–20^ however adjuvant did not further increase the already robust immune response against RABV displayed H_C_50/A.^20^ To increase production and safety of the potential vaccine, we utilized the recombinant rabies virus (RABV) system based on the SAD-B19 strain that has been used previously to stimulate robust immune responses against various pathogens, including Ebola,^21–23^ HIV,^24,25^ anthrax PA,^26^ and BoNT/A.^20^ Particle preparations expressing the H_C_50 antigen from serotypes /A, /B, and /E were found to be immunogenic without the need for adjuvant administration *in vivo* when applied individually or in combination, and the antibody responses were protective in a murine intoxication model.

## Results

### A 30 amino acid extracellular extension of the RABV G is optimal for H_C_50/A expression

**Figure 1:**
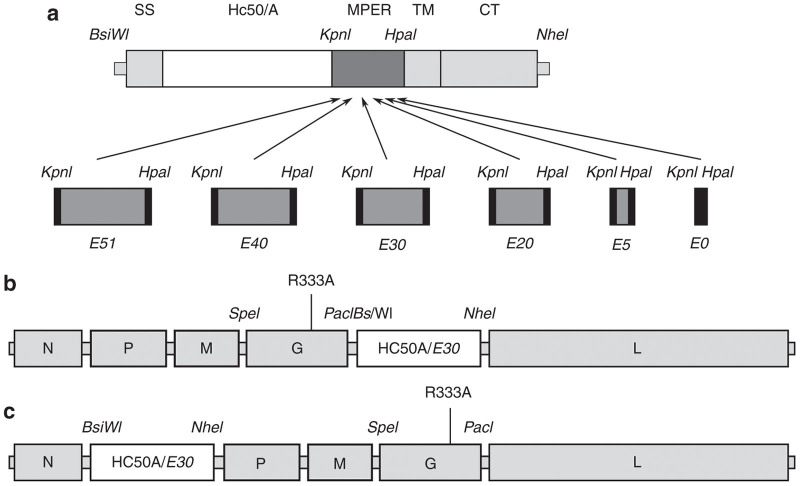
Assembly of recombinant rabies virus (RABV) expressing botulinum neurotoxin type A, B, or E heavy chain carboxyterminal 50 kDa (H_C_50) fragment fused to RABV G ectodomain through cytoplasmic tail. (**a**) The H_C_50 fragment from serotype A was flanked by the RABV G signal sequence (SS) and variable lengths of the membrane proximal extracellular region (MPER, dark grey), transmembrane (TM) and cytoplasmic tail (CT) and restriction sites for insertion into *Bsi*WI and *Nhe*I sites in the RABV genome. (**b**) First-generation H_C_50/A expression vector with R333 mutation. (**c**) Second-generation H_C_50 expression vector with R333 mutation.

As antigen incorporation into viral particles is related to cellular membrane expression levels,^27^ we first sought to confirm the required membrane proximal external region necessary for optimal expression of the serotype A H_C_50 (H_C_50/A) fusion protein by using extensions of 0 (no extension), 5, 20, 30, 40, or 51 amino acids in recombinant rabies virus vectors based on the SAD-B19 vaccine strain (BNSP-333).^28^ These extensions of the membrane proximal external region region were chosen based on our previous observations during the characterization of a RABV vectors expressing anthrax PA^26^ and BoNT H_C_50/A.^20^ In an additional effort to maximize protein expression, we exploited the gradient of transcription associated with the genes of negative strand RNA viruses, where 3′ genes are expressed at the highest levels, and 5′ genes expressed at the lowest levels (the differences between the first (**b**) and second (**c**) generation vectors are illustrated in Figure 1).

BSR cells (a BHK cell clone) were utilized for the initial virus production and analysis. Monolayers of BSR cells were infected at a multiplicity of infection (MOI) of 10 for 48 hours and were analyzed by fluorescence-activated cell sorting (FACS) for recombinant protein expression. We confirmed that the 30 amino acid extracellular extension was optimal for protein surface expression (Table 1). Our phenotypic analysis revealed that there was a nearly twofold increase in surface expression based on mean fluorescence intensity (MFI) of H_C_50/A between the first-generation (SPBN-333-H_C_50/A, MFI = 90.1)^20^ in which the transgene is inserted between the G and L genes and second-generation (BNSP-333-H_C_50/A, MFI = 176) vectors in which the transgene is incorporated between the N and P genes (Figure 1). This was in agreement with previous studies utilizing various nonsegmented negative strand RNA viruses in which additional transcription units were inserted at various locations within the viral genome.^29–31^ Because we sought maximal transgene protein expression, we utilized the 30 amino acid RABV-G membrane proximal external region as the fusion point for the expression of the remaining H_C_50 peptides (Figure 1).

### Codon optimization increases expression of H_C_50/A and /B fusion proteins by recombinant RABV

**Figure 2:**
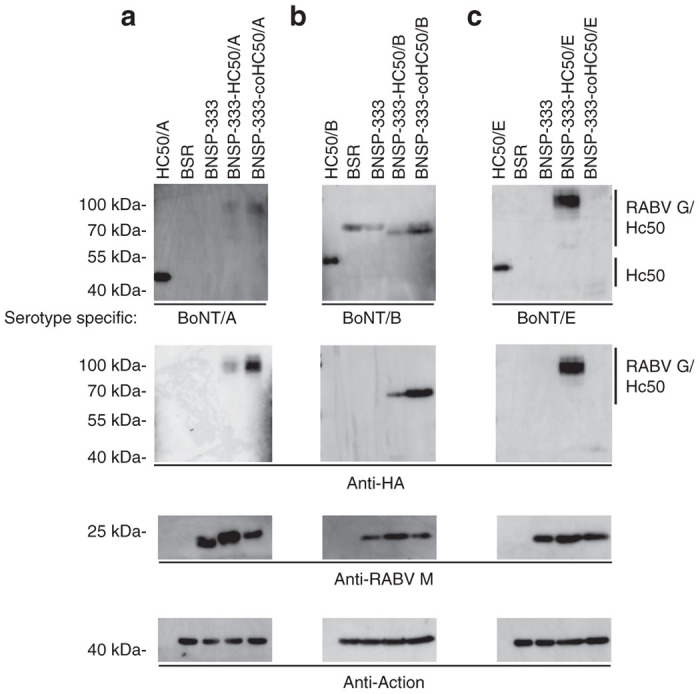
Recombinant H_C_50/RABV-G expression analyses on BSR cells. BSR cells were infected at an multiplicity of infection of 10 for 48 hours with the viruses indicated and probed with (**a**) anti-H_C_50/A1 (NR-9353), (**b**) anti-H_C_50/B1 (NR-9354), or (**c**) anti-BoNT/E1 (NR-17613). Duplicate blots were also probed with anti-HA-tag monoclonal antibody to detect recombinant H_C_50/RABV-G proteins; as infection and loading controls, blots were also probed with anti-RABV-M and anti-β-actin.

As increased levels of surface expression are associated with enhanced incorporation of fusion RABV G/H_C_50 proteins into RABV particles,^27^ we next sought to further maximize protein expression by codon optimizing the different H_C_50 proteins to compensate for mammalian codon bias. Codon-optimized and wild-type versions of the three H_C_50 domains were analyzed by western blot of infected BSR cell lysates. We found that in the cases of serotypes /A and /B, codon optimization resulted in increases in recombinant protein expression (Figure 2a,b, serotype specific and HA specific immunoblots). Although rescued from a cDNA clone with an intact codon optimized H_C_50/E transgene, we observed a loss of expression from the codon optimized H_C_50/E transgene (Figure 2c, serotype-specific and HA-tag-specific immunoblots) by the second passage of the virus. While unexpected, similar observations have been reported previously in which nonsense mutations were acquired in the measles virus F transgene during recombinant VSV replication.^27,32^ In order to determine the mechanism behind the silencing of the codon optimized H_C_50/E transgene, we analyzed the viral RNA and found that there was a frame-shift mutation caused by the incorporation of an additional adenosine residue into an adenosine-rich section of the coding sequence resulting in premature termination (data not shown). While the recombinant RABV G-H_C_50/E protein could be initially detected in the first passage of the virus, this mutation effectively silenced expression of the codon-optimized region by the second passage of the virus (data not shown). We further confirmed that the wild-type BNSP-333-H_C_50/E virus did indeed contain the wild-type gene by sequencing the genomic material from the H_C_50/E high expressing particles (data not shown). Because of these data, we continued to focus on the wild-type H_C_50/E virus.

**Figure 3:**
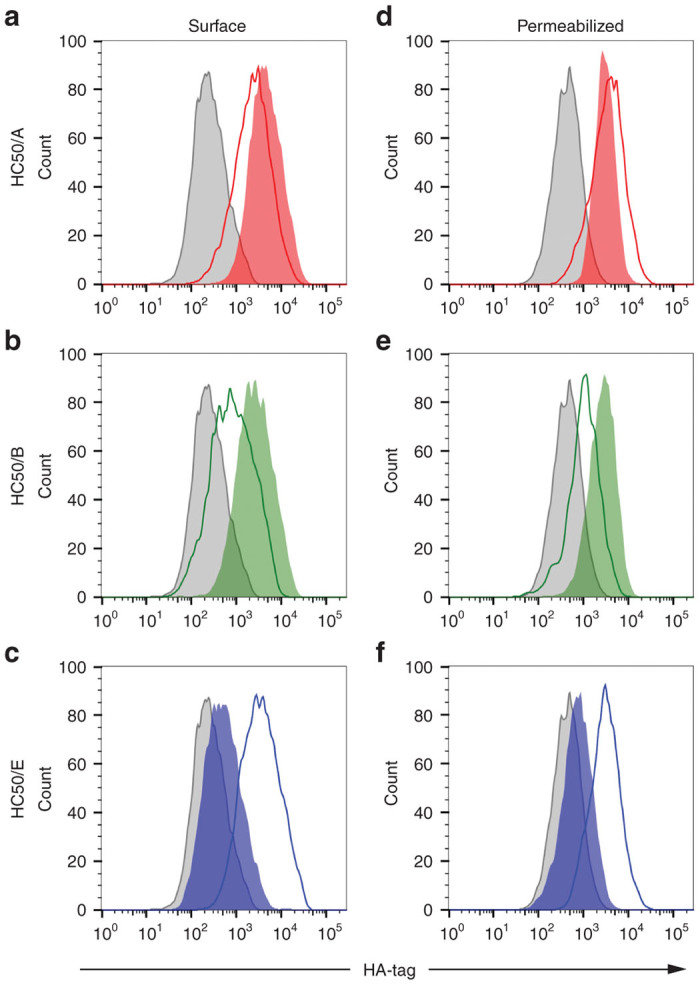
FACS analysis of BSR cells infected with noncodon-optimized or codon-optimized H_C_50 domains. BSR cells were infected at an multiplicity of infection of 5 for 48 hours with BNSP-333 (control, grey), BNSP-333 expressing noncodon-optimized H_C_50 protein (unfilled histograms) or codon-optimized H_C_50 (shaded histograms) and analyzed for surface staining (**a–c**) or total protein (**d–f**) using an anti-HA specific antibody. (**a** and **d**) Experimental samples were infected with BNSP-333-H_C_50/A (red histograms) or BNSP-333-coH_C_50/A (red, shaded histograms). (**b** and **e**) Experimental samples were infected with BNSP-333-H_C_50/B (green histograms) or BNSP-333-coH_C_50/B (green, shaded histograms). (**c** and **f**) Experimental samples were infected with BNSP-333-H_C_50/E (blue histograms) or BNSP-333-coH_C_50/E (blue, shaded histograms).

We also sought to characterize surface expression of the recombinant H_C_50s by FACS analysis. To compare protein expression among the three different serotypes, we utilized a HA-tag specific antibody for the FACS analysis. When comparing cell surface expression between the wild type and codon-optimized viruses, we found that the codon-optimization strategy increased expression of the recombinant proteins for serotypes /A and /B (Figure 3a: MFI of 4,345 versus 2,159, and Figure 3b: MFI of 2,474 versus 728, codon optimized versus wild-type respectively). The results of our western blot analysis of the serotype /E viruses (Figure 2c, top panel) was recapitulated by our FACS analysis (Figure 3f, MFI of 3349 (wild type) versus 496 (codon optimized)), confirming antigen expression from wild type /E and the silencing of the codon-optimized H_C_50/E transgene. In addition, the MFI for wild type /E was also comparable to codon optimized /A and /B (compare Figure 3a–c, MFI of 4,345 [/A], 2,474 [/B], and 3,349 [/E]). Therefore, we focused on the codon-optimized viruses BNSP-333-coH_C_50/A and –coH_C_50/B, and the wild-type sequence –H_C_50/E virus for the remainder of our studies.

### Expression of BoNT-H_C_50 domains from serotypes /B and /E does not reduce virus growth kinetics

**Figure 4:**
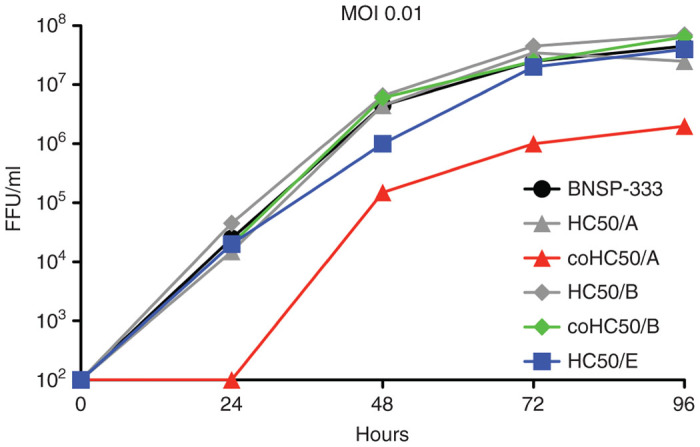
Multistep growth kinetics of recombinant viruses on Vero cells. Vero cells were infected at a multiplicity of infection of 0.01 for 2 hours with BNSP-333 (black), BNSP-333-coH_C_50/A (red), BNSP-333-coH_C_50/B (green), or BNSP-333-H_C_50/E (blue). Samples were collected at the time points indicated and viral titers were determined in duplicate. FFU, fluorescent focus units.

As a potential vaccine product, the growth of recombinant viruses expressing the BoNT H_C_50 domains was analyzed on Vero cells, a continuous cell line approved for vaccine production.^33,34^ We rerecovered the following viruses on Vero cells: BNSP-333-coH_C_50/A, BNSP-333-coH_C_50/B and BNSP-333-H_C_50/E before analyzing their growth on Vero cells. When inoculated at a low MOI (0.01), we observed that there was little difference between the growth profiles of the parental BNSP-333 vector and the BNSP-333-coH_C_50/B or BNSP-333-H_C_50/E viruses (Figure 4). However, we detected an ~1-log difference in peak titers when comparing the parental virus to BNSP-333-coH_C_50/A (Figure 4, compare black circle and red diamond curves). This growth reduction was confirmed with single-step growth kinetics, in which BNSP-333-coH_C_50/A virus again grew to ~1-log lower peak titer compared with the parental virus (data not shown). This contrasts with the analysis of the wild type H_C_50/A expressing virus, which replicated with similar kinetics as the parental virus (Figure 4, compare grey diamond (wild-type H_C_50/A) to black circle curves (parental)). These results indicate that codon optimization of H_C_50/A, while allowing for increasing cell-surface expression (Figure 2a) has a detrimental impact on virus growth kinetics. However, the titer of BNSP-333-coH_C_50/A was still sufficient to produce the vector in the necessary amounts for vaccinations and other studies.

### H_C_50 antigens are incorporated into viral particles

**Figure 5:**
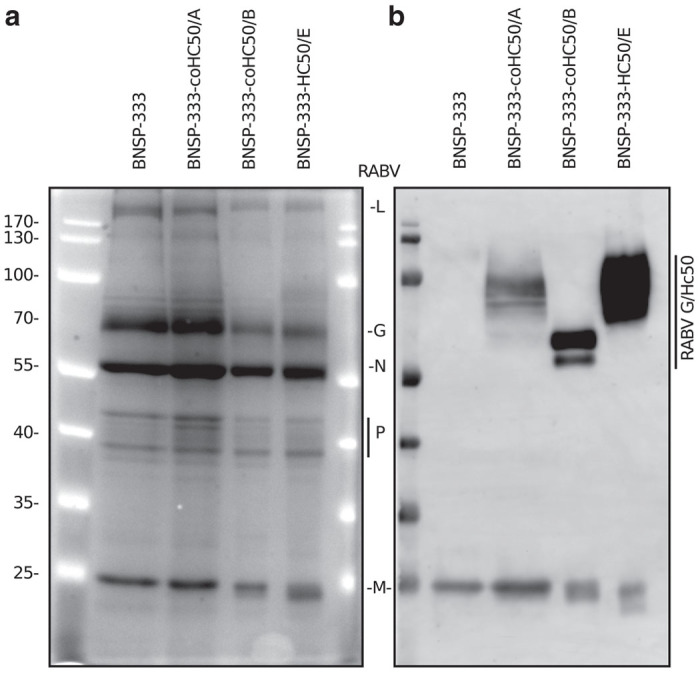
Analysis of H_C_50 incorporation into sucrose-purified particle preparations. (**a**) Sucrose-purified RABV particles (4 µg/lane) were fractioned on 10% sodium dodecyl sulfate–polyacrylamide gel electrophoresis and stained with SYPRO ruby protein stain (RABV proteins; L~242kDa, G~65kDa, N-57kDa, P~38-41kDa, M-25kDa). (**b**) 1 µg of sucrose-purified particles were transferred to polyvinylidene fluoride (PVDF) membranes and probed with anti-HA tag and anti-RABV M-specific antibodies.

We next sought to verify fusion protein incorporation into viruses by analyzing sucrose-purified particles grown on Vero cells. SYPRO Ruby staining of sucrose purified particles showed that the H_C_50/A and /E antigens were incorporated into the viral particle preparations, migrating at just under 100 kDa (Figure 5). The predicted molecular weight of the RABV G-H_C_50/B antigen is ~64 kDa, and comigrates with the RABV G protein and could not be distinguished by SYPRO staining. Western blot analysis using an anti-HA tag analysis of purified particles confirmed H_C_50/B antigen incorporation (Figure 5b).

### Vaccination with RABV expressing the BoNT H_C_50 domains elicits seroconversion in mice

**Figure 6:**
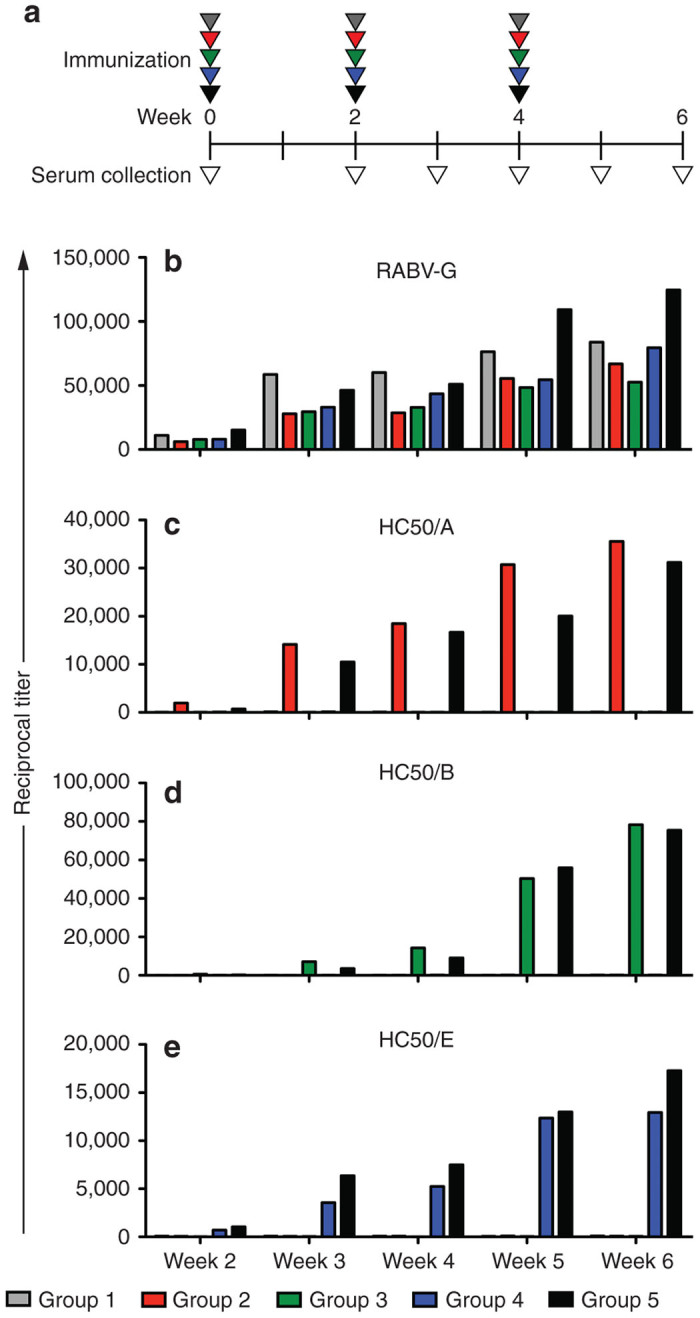
Antibody responses against vector antigens. Sera from vaccinated mice were collected at the time points indicated (**a**) and antibody responses of pooled sera was tittered against (**b**) RABV-G, (**c**) H_C_50/A, (**d**) H_C_50/B, or (**e**) H_C_50/E antigens. Grey bars correspond to the vector control group receiving BNSP-333; red bars correspond to the group receiving BNSP-333-coH_C_50/A; green bars correspond to the BNSP-333-coH_C_50/B group; blue bars correspond to the BNSP-333-H_C_50/E group; black bars correspond to the group receiving the combined particles (BNSP-333-coH_C_50/A, −coH_C_50/B, and −H_C_50/E). Titers are expressed as EC50 values of the reciprocal dilution. The calculated EC50 values represent the effective concentration 50 of the reciprocal dilution (titer) of the sera against each of the groups’ respective antigen(s).

In order to determine the immunogenicity of the selected particles, β-propiolactone (BPL) inactivated particles were used to immunize groups of Balb/c mice (*n* = 5) with 10 µg of either the parental virus particles (BNSP-333, group 1), the selected H_C_50 expressing virus particles individually (/A, /B, or /E corresponding to groups 2–4, respectively), or a mixture of 10 µg of each of the H_C_50 expressing virus particles (group 5), and boosted with the same dose on weeks 2 and 4 (Figure 6a). Immediately before each immunization, serum was harvested from the mice and pooled before assessing seroconversion for RABV-G or the individual H_C_50 antigens. Enzyme-linked immunosorbent assay (ELISA) measurements show that all mice seroconverted for RABV-G within 14 days, at which point the animals had been given a single immunization (Figure 6b). Sera collected every week starting at week 2 were analyzed for reactivity against the BoNT/A, /B, and /E antigens, and demonstrated that the individual serotype immunizations elicited an increasing adaptive immune response over time (Figure 6c,d). Additionally, the combination group (Figure 6, black bars) had titers for each of the individual H_C_50 antigens that were comparable to the individual vector groups (Figure 6c,d, compare groups 2–4 to group 5).

**Figure 7:**
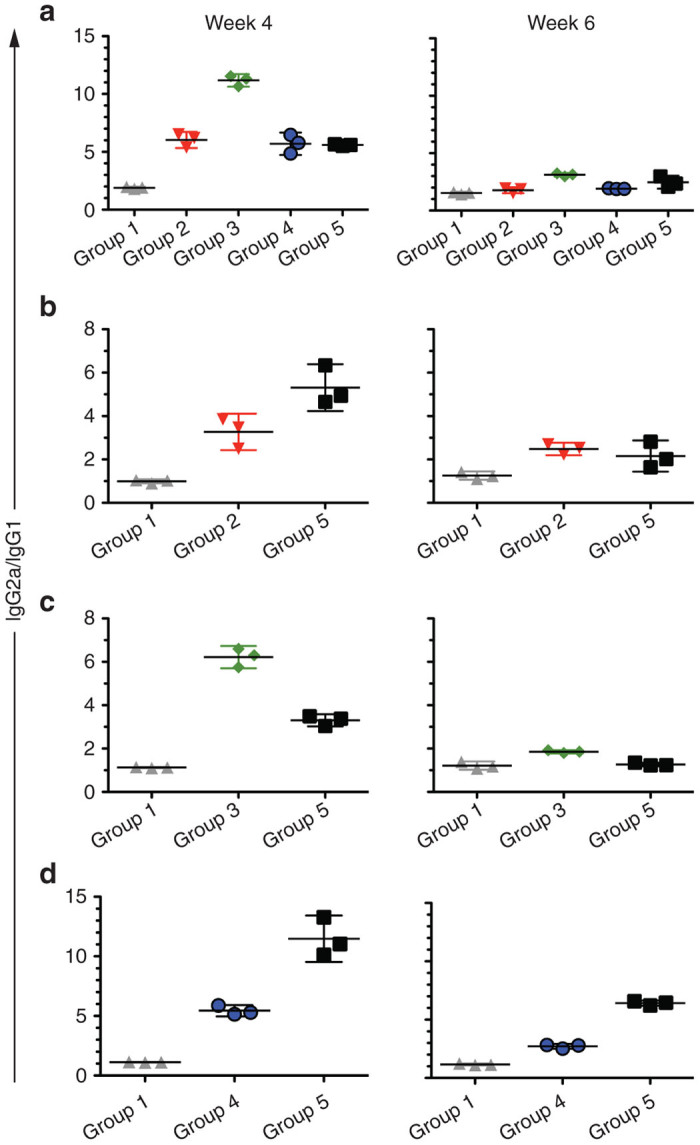
IgG isotype analysis of antibody responses indicates a Th1-biased response. Sera from immunized mice at weeks 4 and 6 were pooled and tested for their IgG1 and IgG2a responses to (**a**) RABV-G, (**b**) H_C_50/A, (**c**) H_C_50/B, and (**d**) H_C_50/E, and presented as the ratio of the optical densities corresponding to the IgG2a to IgG1-specific enzyme-linked immunosorbent assay signals for the specific antigen (each panel represents one antigen). Only the groups with immune responses to the specific antigens, as grouped by panel, were assayed.

To further characterize the immune responses against the different antigens, we analyzed the IgG2a to IgG1 isotype ratio. When the isotype-specific antibody responses from weeks 4 and 6 were analyzed for each of the four different antigens, we found that all groups developed a strong IgG2a (Th-1)-biased response to the H_C_50 antigens by week 4 (Figure 7, left panels). This response, while staying predominantly biased toward a Th-1 like response, had a further increase in IgG1 (Th-2)-specific antibody–antigen responses at week 6 for /A and /B. However, we did not observe a similar increase in the IgG1 response against H_C_50/E in mice immunized with BNSP-333-H_C_50/E particles (Figure 7, right panels, and Supplementary Figure S1).

### Anti-H_C_50 immune responses protect vaccinated mice from lethal BoNT challenge

**Figure 8:**
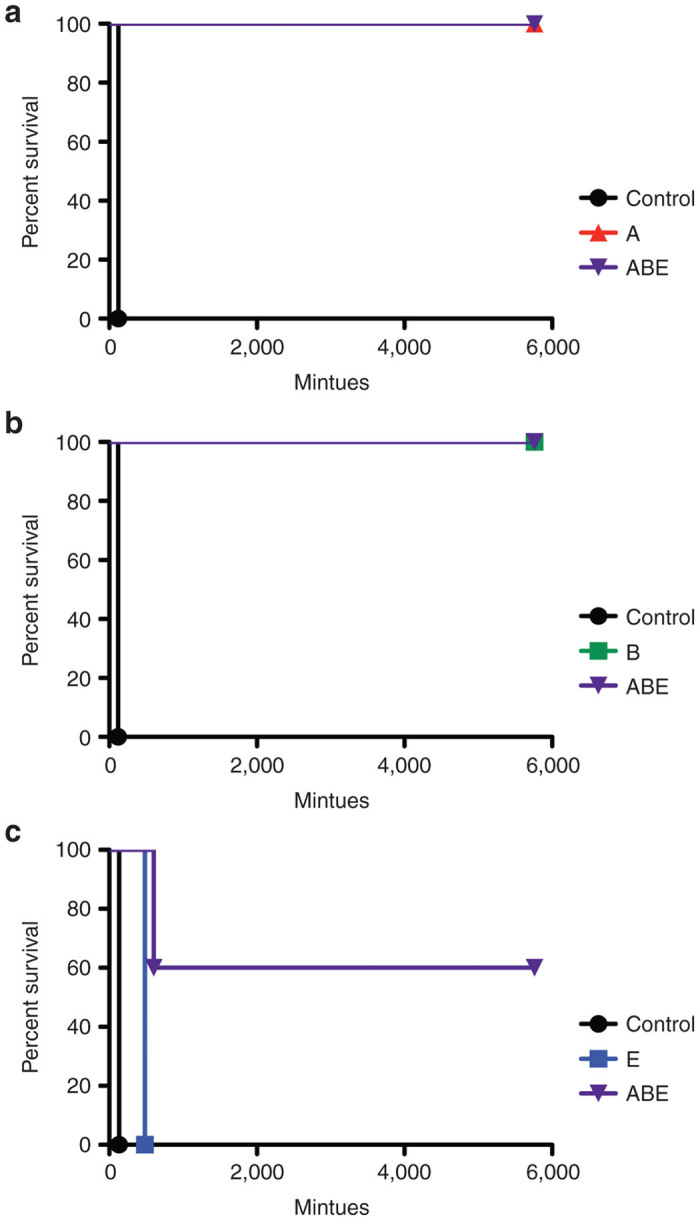
Survival of mice following intraperitoneal challenge with BoNT. Groups of five mice were immunized as outlined in Figure 6a, then challenged via intraperitoneal administration of 1000 mouse LD50 (**a**) BoNT/A, (**b**) BoNT/B, or (**c**) BoNT/E. Combined vaccine groups (ABE) were challenged sequentially with BoNT/A, /B, then /E.

In order to assess the protective efficacy of the immunization schedule, we repeated the immunizations and verified seroconversion (data not shown). These mice were then challenged with 1000 mouse LD50 of the respective BoNTs, a dose sufficient to produce respiratory paralysis within ~120 minutes. A complete (100%) protective response in this model system is survival postchallenge for 4 days. We found that groups of mice (*n* = 5) that were challenged with BoNT/A or /B were completely protected (Figure 8a,b, A and B groups) whereas only a prolongation of survival from 132 minutes for control mice to 480 minutes was seen for mice immunized against serotype /E (Figure 8c; compare black and blue survival curves, *P* = 0.0027). This is noteworthy as it indicates that there is a reduction in toxin potency of between one and two orders of magnitude. All of the mice receiving the trivalent (ABE) immunization were challenged sequentially with BoNT/A, /B, and then /E. The trivalent group survived the first two challenges (Figure 8a,b; ABE group survival curves). When challenged with BoNT/E, three of five mice survived the intoxication (Figure 8c; ABE group survival, *P* = 0.0027 between ABE and control). While immunization with only BNSP-333-H_C_50/E was not able to protect mice from lethal challenge with the cognate toxin, we observed a partially protective response against BoNT/E when incorporated into the trivalent vaccine, indicating some sort of “cross-immunity” or enhanced affect when applied in combination. These data indicate that the multivalent vaccine strategy is effective.

## Discussion

The development of vaccines against botulinum neurotoxins^1^ has historically relied on formaldehyde-inactivated toxoids, which are both dangerous and expensive to produce and may not be optimally immunogenic.^5,35^ Numerous studies have demonstrated the utility of the H_C_50 domain of BoNT in producing a protective immune response.^36–40^ Several groups are investigating alternative strategies for the presentation or delivery of the H_C_50 domains from the various BoNTs. These strategies include the application of recombinant protein or inactivated toxoid, as well as recombinant DNA vaccines.^41^ While each of these approaches have merit, we believe that the application of recombinant viral vectors for vaccine production has several distinct advantages. The first is that by nature, the immunization preparation can serve as a multivalent vaccine, conferring protection against not only the parental virus but against other pathogens as well.^21,28^ In addition to providing protection against multiple pathogens, the preparations themselves can often be administered without the need for additional adjuvants,^20^ and recombinant viruses against important pathogens can be incorporated into pre-established viral vaccine production processes. Moreover, deactivated RABV vaccines are widely produced, administered during both pre- and postexposure prophylaxis and have an excellent safety record.

We have previously shown that RABV expressing the H_C_50/A antigen was effective in protecting mice from a lethal challenge with BoNT/A.^20^ In this study, we sought to enhance and expand our immunization strategy to include protective antigens from two additional BoNT serotypes, /B and /E. We also demonstrate that altering the position of transgene insertion in the RABV genome can affect the expression of the recombinant protein, a phenomenon seen in related viral systems.^29–31^ Expression was further increased by codon optimization, which also increased antigen incorporation into viral particles for two of three serotypes. While focusing on a multivalent immunization strategy, we also investigated the efficacy of the RABV vectors individually. As a potential multivalent vaccine against BoNT and RABV, we chose to maintain the established three-dose immunization scheme during the course of our studies. Following immunization in the trivalent group, we observed protective responses against the corresponding toxins, with complete protection against BoNT/A and /B, and partial protection against BoNT/E. This weaker response against BoNT/E was also observed in the individual H_C_50/E group, both with lower antibody titers and a delay in time until death. This response was observed despite having higher antigen incorporation into the particles compared to the other vectors. Other groups have suggested that an anti-BoNT/E response can be elicited following “hyperimmunization” against BoNT/A-H_C_50.^16,42^ Despite the serological differences that have been utilized to categorize the various BoNTs, our data appear to corroborate this hypothesis since the combined group was afforded a partial protective response in comparison to the individual H_C_50/E group.

Based on the protein characteristics observed during our immunoblot analysis and what has been reported previously, we acknowledge that glycosylation of the H_C_50/E fusion protein could affect the outcome following immunization. Additional studies using *in vitro* transcribed serotype /E heavy chain administered with alum adjuvant also showed only partial effectiveness against BoNT/E challenge.^40^ In addition to the development of lower antibody titers against serotype /E, Zichel *et al*.^40^ also reported that the anti-E response in mice takes longer to develop and requires additional immunizations than the immune responses against either serotype /A or /B. In addition, a similar response has also been observed in humans immunized with the pentavalent ABCDE vaccine.^43^ Meanwhile, other studies have also indicated that glycosylation of the H_C_50 domain can impair the development of neutralizing antibodies against cognate toxin, particularly against BoNT/B, although not necessarily BoNT/A.^5^ In our studies, we observed glycosylation of H_C_50/A but not H_C_50/B when treated with PNGaseF (data not shown); both of these vectors elicited protective responses. Based on the differences in production systems for the H_C_50/E antigen used in our studies (glycosylated) and others (nonglycosylated),^40,43^ it appears that the glycosylation state of the H_C_50/E protein is probably not the major obstacle to /E immunization. Rather, it is more likely that the H_C_50/E protein itself is poorly immunogenic, as seen by the reduced and temporally delayed antibody responses seen during anti-H_C_50/E ELISA titrations.^40^

As we had observed differential responses for the different BoNT serotypes, and since the isotype profile required for neutralizing BoNT has not been investigated previously, we investigated the isotype bias following immunization against BoNT-H_C_50/A, /B, and /E. We initially observed that the immune response against the H_C_50/A and /B antigens was biased toward IgG2a over IgG1, but this bias became less pronounced toward the end of the study timeline. However, in the mice surviving BoNT/E challenge the IgG2a bias was maintained. The importance of biased immune responses has been recently described with anthrax PA.^44^ In their experiments, the ability of antibodies with the same antigen binding domains and heavy chain regions corresponding to IgG1, IgG2a, and IgG2b were assessed for their ability to neutralize the effects of intoxication by intravenous administration of *Bacillus anthracis* Sterne.^44^ Their results indicated a gradient of efficacy, with IgG2a>IgG2b>IgG1 while also requiring a functional Fcγ receptor.^44^ As this system measured the efficacy of antibodies administered prior to IV administration of *B. anthracis,* the dominant protective effects of IgG2a isotype antibodies were not unexpected. These findings were also seen by Varshney *et al*.^45^ where IgG2a isotype antibodies were again more effective during *in vivo* protection assays for staphylococcal enterotoxin B. This is because an IgG2 bias is associated with a Th1 response that results in the activation of macrophages and the production of opsonizing antibodies. Based on these reports and observations, we propose that the isotype biases for the various BoNTs may affect the therapeutic outcome. While future studies will certainly be focused on investigating immunization schedules and strategies that not only maximize antibody titers but also establish complete protection, especially when considering the protection of at risk populations in the event of an act of bioterrorism, a focus should also be directed on the characteristics of protective immune responses. Therefore, in addition to serum titers, particular attention should also be paid to determining the factors influencing isotype biases following immunization (for review, see the commentary by Kozel^46^), which can be used to improve future vaccines.

## Materials and Methods

### Cells and antibodies

BSR (baby hamster kidney) and Vero cells were maintained in Dulbecco’s modified Eagle medium (Mediatech, Manassas, VA) supplemented with 1% penicillin–streptomycin (Mediatech) and 5% fetal calf serum (complete media).

The following reagents were obtained through the NIH Biodefense and Emerging Infections Research Resources Repository, NIAID, NIH: Polyclonal anti-clostridium botulinum neurotoxin A heavy chain fragment (antiserum, goat) NR-9353, polyclonal anti-*C. botulinum* neurotoxin B heavy chain fragment (antiserum, goat), NR-9354 and polyclonal anti-*C. botulinum* neurotoxin E (antiserum, sheep), NR-17613.

### Plasmid construction

Full-length genomic cDNA constructs were based on the pcBNSP-333 backbone described previously.^21,28^ Fusion H_C_50 gene fragments containing a BsiWI restriction site, RABV-G signal sequence (SS), HA-tag, H_C_50 open reading frame from BoNT/A1-Saraburi 2010 (GenBank accession JQ964804), BoNT/B1-Okra (GenBank accession CP000940), or BoNT/E1-CDC47437 (GenBank accession JX424545), then followed by a KpnI restriction site were synthesized by BlueHeron Biotech (Bothell, WA). Human codon optimized fusion genes were also synthesized by BlueHeron. The resultant gene products were ligated into the BNSP-333 vector backbone as outlined in Figure 1. Generation of the various extracellular RABV-G regions has been described previously.^20^ For transient expression, the wild-type and codon-optimized H_C_50/E genes from their respective BNSP-333-based constructs were cloned into the pCG-Fos expression vector (kindly provided by Dr von Messling).^47^

### Virus purification and inactivation

Recombinant RABVs were initially recovered on NA and BSR cells as described previously.^48^ All viruses were then rerecovered on Vero cells as described by Witco *et al*. ^34^ In brief, per six-well plate, a DNA mixture consisting of 5 µg full-length cDNA, 2.5 µg pT7T-N, 1.25 µg pT7T-P, 1.25 µg pT7T-L, 1 µg pT7T-G, and 1.5 µg pCAGGS-T7 were mixed with X-tremeGENE 9 (Roche, Indianapolis, IN) at a ratio of 1:2 and incubated for 15 minutes at room temperature before being added to Vero cells plated the day before at 5 × 10^5^ cells/well. The cells were then incubated in a humidified incubator supplemented with 5% CO_2_ at 34 °C for 3 hours, heat-shocked at 42 °C for 3 hours and returned to 34 °C for 4 days. At such time, the cells were expanded into 60 mm dishes and incubated for an additional 2 days. Afterwards, 1 ml of supernatant was harvested from each of the 60 mm dishes and overlaid on fresh Vero cells and incubated at 34 °C for 2 days before being screened for virus recovery.

Virus stocks were prepared by infecting Vero cells at a MOI of 0.01 in OptiPro medium (Invitrogen, Carlsband, CA) supplemented with 1% penicillin-streptomycin and 4mM L-glutamine (Thermo-HyClone, Rockford, IL). Supernatant harvests were collected at days 3, 6, 9, and 12 postinfection. Supernatants were first concentrated in a stirred cell concentrator (Millipore, Billerica, MA) using a 300 kDa polyethersulfone membrane, then purified by ultracentrifugation at 25,000 rpm for 2 hours at 4 °C over a 20% sucrose cushion in an SW32-Ti rotor (Beckman Coulter, Brea, CA). Pelleted viruses were resuspended in D-PBS (Mediatech) overnight at 4 °C. Viruses were inactivated by incubation with β-propiolactone (Sigma, St Louis, MO) as described previously.^20^

### FACS analysis

For analysis of the various RABV constructs expressing HC50 antigens, BSR cells in six-well cluster plates were infected as indicated in the figure legends for 48 before being stained and analyzed. Following infection, cells were washed 3× with phosphate-buffered saline (PBS) before being detached with Versene and divided equally for total and cell-surface staining. For surface staining, detached cells were incubated on ice with the anti-HA tag antibody (clone HA-7, Sigma) for 1 hour in FACS buffer (PBS supplemented with 10% fetal calf serum and 1% sodium azide). The cells were then washed 3× with FACS buffer, then stained with goat anti-mouse Texas Red (Jackson Immunoresearch, West Grove, PA). Samples were then fixed with 2% paraformaldehyde for 30 minutes at room temperature. For total protein staining, detached cells were fixed for 15 minutes with 2% paraformaldehyde at room temperature, then permeabilized using 0.5% Tween-20 and 2% paraformaldehyde in PBS for 15 minutes at room temperature. Samples were then probed and stained as above. Following a final fixation, samples were resuspended in PBS and sorted using a BD LSR II (Becton Dickinson, Franklin Lakes, NJ) and analyzed using FlowJo vX (Tree Star, Ashland, OR).

### Immunoblot analysis

Western blot analysis was performed on BSR cells infected at an MOI of 10 or transfected with 1 µg pCG-H_C_50/E or codon-optimized pCG-coH_C_50/E for 48 hours before being lysed with RIPA buffer (50 mmol/l Tris, pH 8, 150 mmol/l NaCl, 0.5% sodium deoxycholate, 1% Triton X-100) supplemented with HALT protease inhibitors (Thermo). Total cellular protein (5 µg) was then fractioned by 10% sodium dodecyl sulfate–polyacrylamide gel electrophoresis before transfer to polyvinylidine fluoride membranes using Towbin transfer buffer, then subjected to enhanced chemiluminescence detection using the antibodies indicated in the figure legend.

### Virus growth analysis

Vero cell monolayers in six-well plates were infected at a MOI of either 10 (single-step) or 0.01 (multistep) for 2 hours in serum-free Dulbecco’s modified Eagle medium. The inoculum was then removed and the monolayers were washed three times with PBS to remove unadsorbed virus and the media was replaced with 3 ml complete media. Samples of 100 µl were harvested from cell supernatants at the times indicated and tittered in duplicate on Vero cells.

### Animals and vaccination protocol

Eight to 10-week-old female BALB/c mice (NCI) were immunized by the intramuscular route (i.m.) with 10 µg of either BNSP-333 (parental vector), BNSP-333-coH_C_50/A, BNSP-333-coH_C_50/B, BNSP-333-H_C_50/E, or a combination of serotype /A, /B, and /E vectors (10 µg/each). Mice were boosted twice at 2-week intervals after the initial dose, with blood collected via retro-orbital bleeding prior to initial inoculation, after 2 weeks following each boost and at 5 months postimmunization. The Institutional Animal Care and Use Committee at Thomas Jefferson University approved all animal protocols.

### ELISA

Immulon 4HBX (Thermo) ELISA plates were coated with 50 ng antigen/well (H_C_50/A, H_C_50/E, or RABV-G) or 430 ng H_C_50/B per well in 100 µl coating buffer (5 mmol/l Na_2_CO_3_, pH 9.6), overnight at 4 °C. Plates were then washed three times in PBS-Tween-20 (0.025%, PBS-T) and blocked for at least 1 hour at room temperature with 5% nonfat milk-PBS-T. The plates were then incubated with 100 µl of sera diluted threefold in dilution buffer (PBS supplemented with 0.5% bovine serum albumin and 0.05% Tween-20), overnight at 4 °C. After washing the plates three times with PBS-T, 100 µl horseradish peroxidase–conjugated goat anti-mouse (Jackson ImmunoResearch) in dilution buffer was incubated for 1 hour at room temperature. Plates were again washed three times with PBS-T before being developed with OPD (*o*-Phenylenediamine dihydrochloride) substrate (Sigma), according to the manufacturer’s directions, for 12 minutes. The reactions were quenched by the addition of 50 µl 3 mol/l sulfuric acid and the optical density of the individual wells was recorded at 490 nm using a BioTek EL800 microplate reader (BioTek, Winooski, VT). Data were analyzed in GraphPad Prizm (Version 5.0a, GraphPad Software, La Jolla, CA) and EC50 titers were calculated using the nonlinear regression (curve-fit), Log(agonist) versus response algorithm.

### BoNT neutralization assay

Following the immunization schedule described above, at week 6 botulinum neurotoxin from serotypes /A, /B, and /E, corresponding to 1000 mouse LD_50_ (an amount sufficient to produce respiratory paralysis within a matter of minutes (~120 minutes)) was administered via the intraperitoneal route. Animals were observed throughout the procedure and for five days postinjection and were humanely euthanized in accordance with AAALAC guidelines when obvious signs of neuromuscular weakness were observed. Survival analysis was performed in GraphPad Prizm using the Mantel-Cox test with a 95% confidence interval. All challenge experiments were conducted at the Lankenau Institute for Medical Research and were approved by their institutional animal care and use committee.

## Figures and Tables

**Table 1:** Total expression of fusion HC50/A proteins

*Sample*	*% Live cells^a^*	*Geometric MFI^b^*	*Expression^c^*
Mock	95.3	49.8	47.5
BNSP-333-HC50/A-E0	95.2	63.2	60.2
BNSP-333-HC50/A-E5	85.9	138	118.5
BNSP-333-HC50/A-E20	95.2	132	125.7
BNSP-333-HC50/A-E30	95.4	176	167.9
*SPBN-333-HC50/A-E30*	96.7	90.2	87.2
BNSP-333-HC50/A-E40	96.2	82.9	79.7
BNSP-333-HC50/A-E51	88.6	176	155.9

a Determined by FACS analysis.

b Mean fluorescence intensity (MFI) derived from FACS.

c Expression = (% live cells)*(GMFI).
