# Supplementary figures and images for: Recombinant rabies virus particles presenting botulinum neurotoxin antigens elicit a protective humoral response in vivo
Source: Mol Ther Methods Clin Dev. 2014 Oct 1;1:14046–. doi: 10.1038/mtm.2014.46 (PMC4362357; doi:10.1038/mtm.2014.46)

IgG1

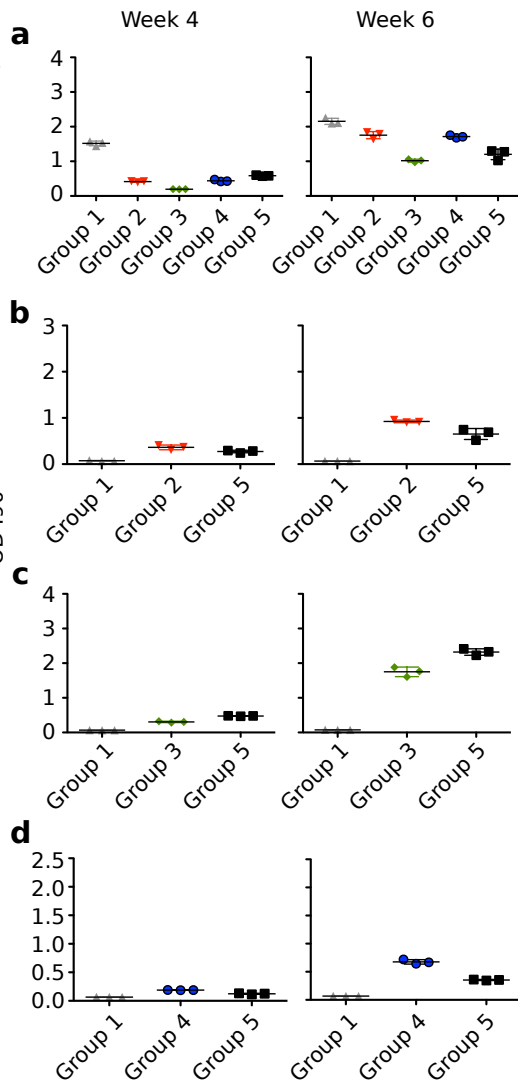

IgG2a

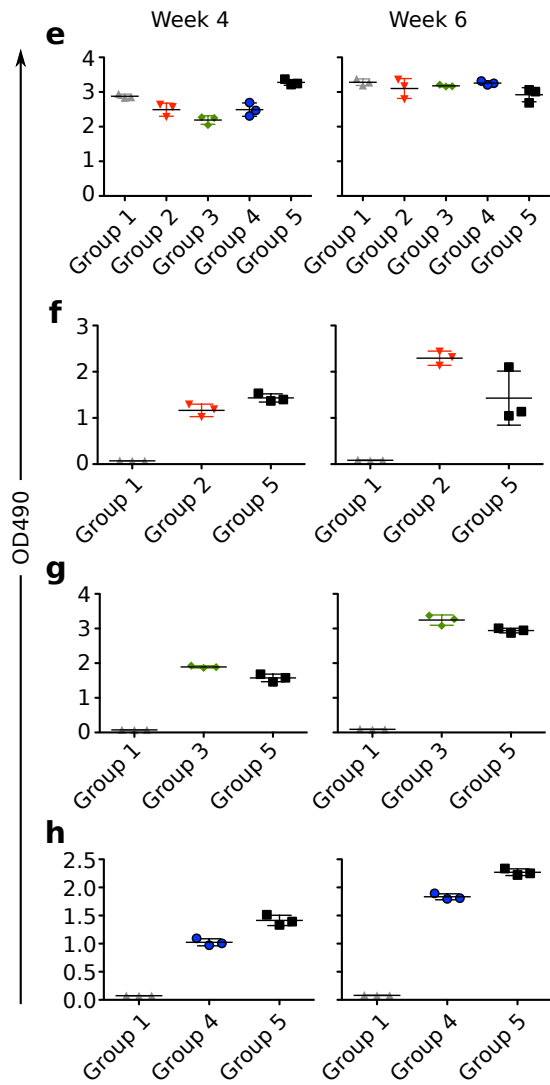

Supplement: Supplementary Figure [file mtm201446-s1.pdf]
